# Sexuelle Gesundheit und Medizin im WIR – Walk In Ruhr: Vorstellung des Zentrums und Ergebnisse der Evaluation

**DOI:** 10.1007/s00103-021-03382-1

**Published:** 2021-07-20

**Authors:** Anja Potthoff, Adriane Skaletz-Rorowski, Sandeep Nambiar, Wiltrud Knebel-Brockmeyer, Andre Kasper, Janet Wach, Arne Kayser, Britta Köhler, Norbert H. Brockmeyer

**Affiliations:** 1 Zentrum für Sexuelle Gesundheit und Medizin, Walk In Ruhr (WIR), Große Beckstraße 12, 44787 Bochum, Deutschland; 2 Interdisziplinäre Immunologische Ambulanz, Zentrum für Sexuelle Gesundheit und Medizin, Klinik für Dermatologie, Venerologie und Allergologie, Ruhr Universität Bochum, Bochum, Deutschland; 3 Gesundheitsamt Bochum, Bochum, Deutschland; 4 Aidshilfe Bochum e. V., Bochum, Deutschland

**Keywords:** Multiprofessionelle Versorgung, HIV/STI, Prävention, Diversität, Health Adviser, Multiprofessional care, HIV/sexually transmitted infections, Prevention, Diversity, Health adviser

## Abstract

**Hintergrund:**

Ein ganzheitliches Konzept sexueller Gesundheit und Medizin berücksichtigt die Diversität von Lebenswelten, um Klient*innen für Prävention, Testung, Beratung und Behandlung sexuell übertragbarer Infektionen (STI) zu gewinnen. Mit diesem Verständnis arbeiten die Immunologische Ambulanz, das Gesundheitsamt, die Aidshilfe sowie weitere Selbsthilfeorganisationen im WIR – Walk In Ruhr, Zentrum für Sexuelle Gesundheit und Medizin, in Bochum zusammen.

**Ziel der Arbeit:**

Am Beispiel des WIR, das mit seinem innovativen Konzept eines Versorgungszentrums multiprofessionell innerhalb eines Settings sektor- und rechtsformübergreifend arbeitet, wird gezeigt, dass Erreichbarkeit, Test- und Behandlungsquote und HIV/STI-Risiko-Selbsteinschätzung sich verbessern. Das WIR wurde zudem im Auftrag des Bundesministeriums für Gesundheit über 3 Jahre extern evaluiert auch diese Ergebnisse werden dargestellt.

**Methode:**

Das Konzept des WIR und die im WIR durchgeführten Studien werden deskriptiv dargestellt. Bei der externen Evaluation wurde ein Mixed-Method-Design aus quantitativen und qualitativen Erhebungen angewandt.

**Ergebnis:**

Durch die Kooperation werden häufiger Frauen (27,7 %) und Heterosexuelle (56,4 %) als in der Ambulanz allein erreicht. Die Rate positiver Testergebnisse im WIR stieg von 2017 bis 2018 von 9,3 % auf 12,6 %.

**Diskussion:**

Durch das integrative Versorgungskonzept des WIR gelingt es, Menschen mit HIV/STI früh zu erreichen und zu behandeln. Der Übergang von Prävention zu medizinischer Versorgung ist im WIR von zentraler Bedeutung. Gesundheitsberater sind ein wichtiges Instrument für die aufsuchende Arbeit. Psychosoziale sowie psychotherapeutische Beratung werden in hohem Maße genutzt. Durch die vielschichtigen Angebote von Prävention, Test, Beratung werden bessere medizinische Ergebnisse erreicht sowie die Eigenverantwortung für sexuelle Gesundheit gesteigert. Die Übertragung des Konzeptes in die Fläche kann einen Beitrag zur besseren Versorgung zu sexueller Gesundheit leisten.

## Abstract (en)

**Background:**

Holistic sexual healthcare factors in diversity of social habitat and aims to improvise client outreach for prevention, testing, counseling, and treatment of STIs. Towards this goal, the immunology outpatient clinic, the public health department of Bochum, the AIDS Service Organization Bochum e. v., and other community-driven NGOs mutually cooperate under the umbrella of WIR – Walk In Ruhr, Centre for Sexual Health and Medicine.

**Objectives:**

WIR is an innovative concept for multi-professional in-house ambulatory healthcare with cross-sectoral and cross-legal reach. It has successfully improved accessibility, testing and treatment rates, and HIV/STI self-assessment. We present the results achieved at WIR.

**Methods:**

A mixed-method design of qualitative and quantitative surveys.

**Results:**

The WIR reaches more women (27.7%) and heterosexuals (56.4%) than other counseling/test centers. The rate of positive test results at the WIR increased from 9.3% in 2017 to 12.6% in 2018 and progress from prevention to medical care is a significant aspect of WIR. The Federal Ministry of Health has externally evaluated WIR for over three years.

**Discussion:**

The integrative care model of WIR allows for early outreach and treatment of individuals with HIV/ST infections. Health advisors remain an important instrument facilitating outreach and psychosocial/psychotherapeutic counseling is administered frequently. Such a multi-layered approach in prevention, testing, and consultation, leads to improvement in both medical outcomes and the self-responsible attitude of patients towards their sexual health. Hence, expansion of integrative care models like WIR on a wider scale could arguably contribute to better health service and sexual health.

## Einleitung

„Jeder Mensch kann im Laufe seines Lebens mit einer sexuell … übertragbaren Infektion in Kontakt kommen. Daher gilt es, dem Lebensalter und den Lebensumständen entsprechende Angebote zugänglich zu machen, um Infektionen einzudämmen und individuelle und gesellschaftliche Auswirkungen zu minimieren.“ Dieses Zitat stammt aus der „Strategie zur Eindämmung von HIV, Hepatitis B und C und anderen sexuell übertragbaren Infektionen“ der Bundesregierung BIS 2030 („BIS 2030“; [1]). Die hier geforderten Angebote gehen über rein aufklärende, präventive und kurative Versorgungsstrukturen hinaus. Explizit wird die Berücksichtigung von Lebenswelten (persönliches Umfeld) als Voraussetzung eingestuft, um die obigen Infektionen zu reduzieren und damit Folgen für den Einzelnen und die Gesellschaft zu verringern.

Um ein solches innovatives sozialmedizinisches Versorgungskonzept umzusetzen, wurde im Jahr 2016 in Bochum das WIR – Walk In Ruhr, Zentrum für Sexuelle Gesundheit und Medizin, gegründet. Multiprofessionalität wurde erzielt durch die Zusammenarbeit verschiedener Einrichtungen: der Interdisziplinären Immunologischen Ambulanz, des Zentrums für Sexuelle Gesundheit und Medizin, der Klinik für Dermatologie, Venerologie und Allergologie, Ruhr Universität Bochum, des Gesundheitsamts (GA) Bochum, der Aidshilfe (AH) Bochum e. V. sowie Madonna e. V., Pro Familia e. V. und Rosa Strippe e. V. Das Zentrum arbeitet sektor- und rechtsformübergreifend, hat eine interdisziplinäre Verweisungskompetenz mit kurzen Wegen unter einem Dach. Als Erstkontakt bieten Gesundheitsberater (Health Adviser, HA) Gespräch und Beratung an und begleiten, je nach festgestelltem Bedarf, die Überleitung in die jeweilige Institution. In BIS 2030 [1] schreibt das Bundesgesundheitsministerium (BMG) weiter: „Für die Umsetzung der Strategie müssen alle relevanten Akteure ebenenübergreifend zusammenarbeiten. Bund, Länder, kommunale Selbstverwaltung, Öffentlicher Gesundheitsdienst, freie Träger, die Selbsthilfe, Ärzteschaft, Pflegekräfte sowie die Bereiche Justiz, Bildung und Arbeit sind gefordert.“

Die Beratungs‑, Test- und Behandlungsangebote bei Infektionen mit HIV (humanes Immundefizienzvirus) und anderen sexuell übertragbaren Infektionen (STI) werden durch psychotherapeutische und psychosoziale Angebote ergänzt, um einen umfassenden Beitrag zur sexuellen Gesundheit im Sinne der Definition der Weltgesundheitsorganisation (WHO; [2]) wie auch der Deutschen STI-Gesellschaft (DSTIG e. V.) zu leisten. Ziel des WIR ist, Menschen darin zu bestärken, Präventions‑, Test- und Versorgungsangebote wahr- und anzunehmen.

**Figure 1:**
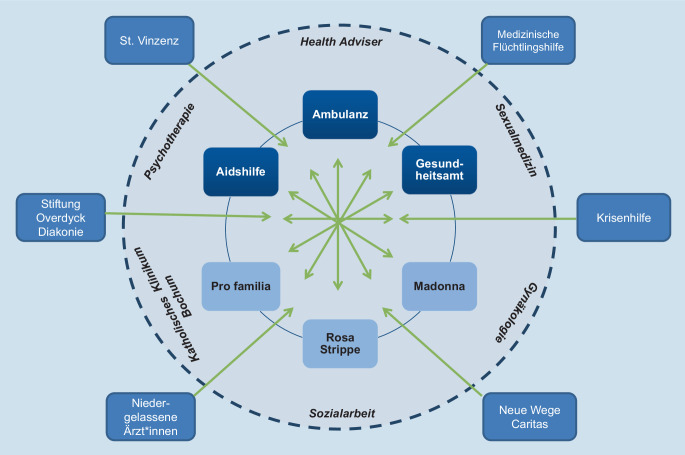

Eine niederschwellige Willkommensstruktur mit akzeptierender Grundhaltung zu diversen Lebenswelten ist im WIR Voraussetzung, um Individuen zu erreichen. Leitgedanke des Zentrums ist, nicht nur Verhaltensprävention, sondern auch Verhältnisprävention umzusetzen (Abb. 1).

Mit dieser Arbeit soll gezeigt werden, dass Verhaltensprävention und Verhältnisprävention, die im WIR umgesetzt werden, zu einer größeren und breiteren Akzeptanz und Erreichbarkeit der Klient*innen führen, Angebote zur sexuellen Gesundheit und Medizin anzunehmen. Die Publikation soll am Beispiel des WIR zeigen, dass multiprofessionelle Versorgung in einem Zentrum nicht nur zu kürzeren Wegen, sondern zu einer schnelleren, umfassenderen medizinischen, psychosozialen und -therapeutischen Versorgung führt. Auch soll sie einen Überblick über innovative Angebote im WIR und deren jeweilige Relevanz geben. Anhand von Beispielen, welche Kooperationen und Netzwerke mit dem WIR aufgebaut werden konnten, soll demonstriert werden, wie wichtig es ist, dieses und ähnliche Versorgungskonzepte in die Fläche, beispielsweise in unterversorgte ländliche Bereiche, zu übertragen.

Im Folgenden wird zunächst die Rolle des WIR bei der Eindämmung von STI unter Berücksichtigung der besonderen Versorgungsproblematik von symptomlosen STI beschrieben. Danach werden die medizinischen Schwerpunkte und Angebote des Zentrums inklusive erhobener Daten zur Nutzung der Angebote vorgestellt. Es folgen Hintergrundinformationen und Ergebnisse der 3‑jährigen Projektevaluation im Auftrag des BMG und weiterer wissenschaftlicher Studien und Projekte, an denen das WIR beteiligt ist. Die Diskussion setzt sich damit auseinander, welche Einflüsse mit welcher gesundheitspolitischen Relevanz die Angebote und Projekte des WIR auf die sexuelle Gesundheit und deren Versorgung haben.

## Die Rolle des WIR bei der Eindämmung von STI

HIV ist in der Öffentlichkeit sehr bekannt [3]. Neben der Regelversorgung von Menschen mit HIV-Infektion ist es ein Ziel des WIR, die von den Vereinten Nationen für HIV/Aids (UNAIDS)^1^ gesetzten Ziele umzusetzen: 95 % der HIV-Infizierten sind diagnostiziert, 95 % therapiert, bei 95 % ist HIV im Blut nicht nachweisbar (und daher sind diese nicht infektiös). Im WIR wurde das Ziel „Therapie als Prävention“ erreicht, der Anteil der Patienten mit nicht nachweisbarer HIV-Viruslast konnte von 94 % (2018) [4, S. 149] auf 98 % (2020) gesteigert werden (nicht publizierte Daten Jahresbericht).

Andere STI sind weit weniger oder teils unbekannt, obwohl sie einen größeren Teil der Bevölkerung betreffen als HIV [3]. Beratungen von Gesundheitsamt (GA) und HA machten deutlich, dass bei Klient*innen häufig der ausschließliche Wunsch nach einem HIV-Test besteht, obwohl das Risiko für andere STI größer ist. Bei einem integrierten Angebot von HIV-, STI-Tests und -Beratung kann ein erheblicher Anteil auch asymptomatischer STI diagnostiziert, therapiert, Infektionsketten durchbrochen und Strategien zum Risikomanagement entwickelt werden. Die Zahl der positiven STI-Diagnosen steigt in den letzten Jahren in Deutschland und anderen Ländern deutlich an [5–9]. Dies liegt zum einen an veränderten sexuellen Verhaltensweisen und Einstellungen, wie z. B. der erhöhten, weltweiten sexuellen Mobilität [10]. Zum anderen haben die Möglichkeiten zu sexuellen Kontakten durch Internetkontaktportale deutlich zugenommen. Hinzu kommt, dass HIV-Patient*innen – unter Therapie nicht mehr infektiös – sowie Klient*innen mit Präexpositionsprophylaxe (PrEP) keine Kondome nutzen müssen, aber als Folge STI häufiger auftreten.

Da STI häufig asymptomatisch verlaufen, ist es notwendig, zum einen bei Klient*innen das Wissen um Risikoverhalten zu erhöhen, sodass sie ihr eigenes Sexualverhalten einschätzen können, um längerfristig ein risikoadaptiertes Verhalten zu fördern. Zum anderen wird das Bewusstsein gefördert, entsprechend ihres individuellen Risikoverhaltens regelmäßige Testungen und ggf. Behandlungen wahrzunehmen. Da STI mit Schamgefühlen und Stigma verbunden sind, bietet das WIR einen offenen und tabufreien Dialog, mit Akzeptanz von Vielfalt und Selbstbestimmung und setzt die Stärkung der Eigenverantwortung als wesentliches Prinzip seiner Arbeit entgegen.

Im WIR arbeitet ein multiprofessionelles Team aus ärztlichen Fachkräften für Dermatologie, Infektiologie, Proktologie und Gynäkologie, medizinischen Fachangestellten, Psychotherapeut*innen, Psycholog*innen, Sozialarbeiter*innen und HA. Hinzu kommen Fachkräfte für Öffentlichkeitsarbeit sowie wissenschaftliche Mitarbeiter*innen für die Konzeptionierung und Durchführung von Projekten und Studien. Ähnliche multiprofessionell ausgerichtete Einrichtungen existieren aktuell besonders im angelsächsischen Raum [11].

Der Beruf der HA ist 1947 als Reaktion auf die Syphilisepidemie in den USA eingeführt worden und im Vereinigten Königreich (UK) ein wichtiger Teil der weitverbreiteten Sexual Health Clinics. Die HA sind in einem national agierenden Berufsverband – der Society of Sexual Health Advisers (SSHA) – vernetzt. In Deutschland ist der Beruf weitgehend unbekannt. Im WIR sind HA seit 2016 fester Bestandteil der Versorgungsstruktur und mit dem SSHA vernetzt. Die geschulten Mitarbeiter*innen (z. B. Pflege, Hebammen, Hochschule für Gesundheit) werden im WIR curricular fortgebildet. Das Curriculum wurde im WIR konzipiert [12]. Als Mittler zwischen Klient*innen und den Institutionen des WIR sind die Aufgaben von HA:

- Erkennen der Klient*innenbedarfe im Erstkontakt,
- Lotsen innerhalb der Institutionen,
- Ansprechpartner*innen in psychosozialen Fragen,
- aufsuchende Arbeit, z. B. Schulen, Partyszene, wohnungslose Jugendliche, und
- Testangebote vor Ort.

In den Jahren 2018 und 2019 konnten die HA zusätzlich zu den in der Leistungsdokumentation des Evaluationsberichtes erfassten 433 Fällen, im WIR mehrere Tausend Menschen erreichen (u. a. ca. 2000 Klient*Innen im WIR und ca. 700 Schüler*innen und mind. 600 Kund*innen von Swingerclubs im Rahmen von aufsuchender Arbeit [13, Poster DSTIG 2021]). An der Patientenstruktur wird erkennbar, dass auch besonders vulnerable oder schwer zu erreichende Personengruppen durch die aufsuchenden Angebote der HA für eine Behandlung im WIR gewonnen werden. So heißt es im Evaluationsbericht: „Zwischen Januar 2017 und Juni 2019 fanden deutlich über 200 Aktionen statt, vor allem an Schulen und Jugendeinrichtungen. Einige Angebote waren an (minderjährige) Flüchtlinge gerichtet. Gänzlich neu entwickelt wurde die Ansprache in Swingerclubs“ [4, S. 171].

## Medizinische Schwerpunkte und Angebote des WIR

Wesentliche Voraussetzung für die Annahme medizinischer Angebote ist die Kommunikation zwischen Patient*innen und Behandler*innen („sprechende Medizin“), die unerlässlich ist, um wichtige Themen anzusprechen, wie:

- sexuelle (unerfüllte) Orientierung und sexuelle Vorlieben,
- Strategien der Risikoreduktion in Hinblick auf STI, Safer Sex und Safer Use,
- Kondom-(nicht‑)Nutzung und
- Substanzkonsum in Zusammenhang mit sexuellem Risikoverhalten (Chemsex).

Nur im offenen und tabufreien Dialog – zwischen Behandler*in und Klient*in – kann Eigenverantwortung für sexuelle Gesundheit vermittelt werden.

Im WIR gibt es eine große Auswahl medizinischer Angebote, u. a.:

- Diagnostik und Therapie von STI (auch ohne Krankenversicherung),
- Kontrolle des Therapieerfolges, Resistenztestungen,
- Partner*innen-Mitbehandlung und -Benachrichtigung,
- Impfungen,
- Test- und Therapiemöglichkeiten im Rahmen von Studien,
- Beratung und Unterstützung zu Therapieadhärenz,
- Überleitung zu psychosozialer Beratung,
- Psychotherapie bei psychischen Störungen (Trauma), bei Substanzkonsum,
- Beratung und medizinische sowie psychosoziale Begleitung der PrEP,
- gynäkologische Versorgung für Menschen ohne Regelversorgung oder Stigmaerfahrung,
- Spezialsprechstunden, u. a. für Jugendliche und für Menschen mit Fragen zu Transsexualität.

*Ambulante Tests* werden von den HA des WIR für schwer erreichbare oder vulnerable Menschen wie wohnungslose Jugendliche, für Sexarbeiter*innen in Bordellen und Clubs sowie in der „Lifestyleszene“ (Swingerclubs) angeboten. Ziel der *aufsuchenden Arbeit* ist, aufzuklären über sexuelles Risikoverhalten, das Angebot zum Test zu machen und ein Bewusstsein der Eigenverantwortung für die sexuelle Gesundheit und für die Gesundheit im Allgemeinen zu schaffen.

Zu den Aufgaben der HA gehört auch die *Jugendarbeit zu sexueller Gesundheit*, bspw. der Besuch von Schulen und Jugendhilfeeinrichtungen, Workshops in der Flüchtlingshilfe, bei Jugendorganisationen wie Pfadfindern, oder Bildungsgänge im Justizvollzug. Ziel ist immer, das Verantwortungsbewusstsein für die eigene Gesundheit zu stärken, durch Informationen und Gespräche zu Sexualität und sexueller Gesundheit, die im Elternhaus, in der Schule und teils auch in den Peer Groups häufig wegen Scham oder Tabus nicht möglich sind. In den Jahren 2018 und 2019 wurden bspw. jeweils über 700 Schüler*innen verschiedenster Schulformen und Altersgruppen in Bochum und Umgebung über sexuelle Gesundheit informiert.

Der große Bedarf an *psychosozialer Beratung* wird im WIR zum großen Teil von der Aidshilfe (AH) geleistet. Auch die HA versuchen, die Notwendigkeit einer psychosozialen Beratung zu erkennen, sie anzubieten und die Klient*innen zeitnah in die jeweilige Institution zu vermitteln.

Es wird angeboten:

- zielgruppenspezifische Beratung für junge Menschen (Jugendsprechstunde) oder zu altersspezifischen Problemen,
- Unterstützung bei Behörden: Krankenversicherung, Rentenanträge u. a.,
- Gesundheits- und Sozialberatung von Sexarbeiterinnen,
- sozialrechtliche Beratung von Männern und Frauen mit HIV,
- Hilfestellungen zu betreutem Wohnen,
- Ein- oder Ausstiegsberatung für Prostituierte,
- Beratung zu geschlechtlicher Identität,
- Fragen zu suchtbezogenen Optionen u. v. m.

Im WIR steht die psychosoziale Beratung meist im Zusammenhang mit der medizinischen Versorgung, so beispielsweise mit:

- Medikamentenadhärenz, Therapietreue,
- Präventionsmöglichkeiten, hier auch PrEP,
- Testangeboten, auch anonym.

Im Jahr 2018 wurden im WIR insgesamt 3901 psychosoziale Leistungen dokumentiert. In diesem Zeitraum wurden 880 Nutzer*innen, 34 % der WIR-Klientel erreicht. Die AH dokumentierte für 88 % ihrer Klient*innen psychosoziale Leistungen, die Ambulanz für 35 % und das GA für 17 % der Nutzer*innen [4].

Ein Teil der Besucher*innen des WIR leidet an einer psychischen Störung mit Krankheitswert. Eine Überleitung zur Krisenintervention erfolgt möglichst direkt. Die Wartezeit auf ein Erstgespräch beträgt wenige Tage. Typische Anlässe für eine *psychotherapeutische Intervention* sind: eine akute emotionale Krise (z. B. infolge einer HIV-Erstdiagnose), Trauma, Ängste, affektive Störungen, problematischer Drogen- und/oder Alkoholkonsum, Borderlinestörungen mit einhergehendem, dauerhaft riskantem Sexualverhalten u. a. Im WIR werden auch Menschen ohne Zugang zu einer klassischen psychotherapeutischen Versorgung, z. B. wegen Drogen- und Alkoholkonsums, behandelt.

Das WIR hat weiter Angebote, die speziell für das Zentrum entwickelt wurden: ein Online-HIV/STI-Risikotest, die Partner*innen-Benachrichtigung mit Onlinetool und das Probenselbstentnahmekit „TeST-It“. Mithilfe des im WIR konzipierten *Online-HIV/STI-Risikotests* erhalten die Nutzer*innen eine grobe Einschätzung ihres HIV- und STI-Risikos [14]. Im Laufe des Tests werden, auf die individuellen Antworten bezogen, Hinweise zu Schutz- und Testmöglichkeiten gegeben. Diese werden bewusst während des Tests und nicht zum Ende des Fragenkatalogs angezeigt, um auch bei jenen Menschen Präventionsbotschaften zu platzieren, die den Test nicht bis zum Ende durchführen. Der Test dient zudem als Türöffner, um Menschen für sexuelle Gesundheit zu interessieren. Der Test wird wissenschaftlich begleitet (Ethiknummer: 17-6208_2-BR). Seit Juni 2017 bis Mai 2020 haben 20.387 Nutzer*innen den Test angeklickt. Davon haben 11.529 Nutzer*innen den Test vollständig absolviert.

Die Partner*innen-Benachrichtigung mit Onlinetool soll STI-Infektionsketten unterbrechen. Die Partner*innen-Benachrichtigung ist immer freiwillig. Da nicht jede*r seine/ihren Partner*innen persönlich informieren möchte, hat das WIR (Juni 2017) ein Onlinetool entwickelt, mit dem bis zu 15 Sexualpartner*innen per E‑Mail oder SMS anonym erreicht und über eine bestehende STI der Kontaktperson informiert werden können, verbunden mit dem Angebot zu Beratung, Testung und ggf. Therapie. Der Absender ist das WIR [15]. Gespräche zur Partner*innen-Benachrichtigung führen alle beteiligten Berufsgruppen im WIR. Es wird eine Partner*in-Mitbehandlung angeboten und ermöglicht. Der Therapieerfolg wird kontrolliert, auch um Resistenzentwicklungen vorzubeugen. Bis Ende 2020 haben 327 Klient*innen die anonyme Partner*innen-Benachrichtigung genutzt.

Seit Herbst 2018 ist es möglich, das vom WIR und der AH Nordrhein-Westfalen (NRW) entwickelte *Probenselbstentnahmekit „TeST-It“* zu erwerben und die zu Hause entnommenen Proben im Labor auf die 5 wichtigsten STI testen zu lassen. Mittels eines beiliegenden Fragebogens werden Daten zum Sexualverhalten und zur Demografie der Nutzer*innen erfasst und wissenschaftlich ausgewertet (Ethiknummer: 17-6208_2-BR). Durch ein Pseudonym wird sichergestellt, dass die Mitteilung des Ergebnisses über die Kontaktperson des WIR erfolgt. Gerade in ländlichen Orten können damit Klient*innen den häufig mit Scham behafteten Wunsch nach einem STI-Test umgehen.

Im WIR werden auch *Fortbildungen* angeboten. Defizite in der Ausbildung für Gesundheitsberufe hinsichtlich der Berücksichtigung von STI und besondere Erfordernisse in diesem Bereich führten dazu, dass in Zusammenarbeit mit der DSTIG e. V., der Bundeszentrale für gesundheitliche Aufklärung (BZgA) und der Ärztekammer Westfalen-Lippe ein „Curriculum Sexuelle Gesundheit und STI“ entwickelt wurde [12]. Die Öffnung dieser Fortbildung [12] auch für nichtärztliche Teilnehmer*innen (TN) war ein wichtiger Schritt, um der Interdisziplinarität dieses Arbeitsgebiets adäquat gerecht zu werden. Seit Einführung 2017 haben in 6 Durchgängen bereits 168 Personen dieses Angebot wahrgenommen, was das Interesse, aber auch den Bedarf belegt.

Jährlich wird der Fachtag „Sexualität und Psyche“ vom WIR sowie vierteljährlich in Zusammenarbeit mit niedergelassenen Ärzt*innen der „Qualitätszirkel HIV, STI, Hepatitis und andere Infektionen“ angeboten. Studierende werden bei Pflichtpraktika, Famulaturen, Master- und Doktorarbeiten begleitet.

Um auch schwerer zu erreichenden Menschen den Weg ins WIR zu bahnen, wurden *Kooperationen mit Organisationen* aufgebaut: St. Vinzenz e. V. (Jugendhilfeeinrichtung), Diakonie (Evangelische Stiftung Overdyck – Kinder‑, Jugend- und Familienhilfe), Krisenhilfe e. V., Neue Wege Caritas (Beratungsstelle gegen sexuellen Missbrauch von Kindern), Medizinische Flüchtlingshilfe e. V. und weitere (Abb. 1).

## Wissenschaftliche Arbeit des WIR

### *Externe Evaluation im Auftrag des BMG*

Das WIR wurde im Auftrag des BMG durch die Institute FOGS GmbH und delphi GmbH evaluiert, beratend unterstützt durch das Zentrum für Infektionsforschung, Uniklinik Hamburg-Eppendorf. Die Umsetzung des Evaluationskonzepts wurde vom WIR unterstützt, so wurden Leistungs- und Tätigkeitsdokumentationen von WIR-Mitarbeiter*innen geführt und die Durchführung von Gesprächen mit verschiedenen Akteursgruppen organisiert. Auch die vom WIR dokumentierten klinischen Daten wurden für die statistische Auswertung durch FOGS und delphi zusammengestellt.

Als Methode wurde bei der Evaluation ein Mixed-Method-Design angewandt, das quantitative Erhebungen mit qualitativen Bausteinen kombinierte (teilnehmende systematische Beobachtungen, qualitative Interviews und Fokusgruppen, Sekundäranalysen, Nutzer*innenbefragung, Dokumentation von Klient*innen‑, Leistungs- und Tätigkeitsdaten) und die Sichtweisen aller Akteursgruppen (Leitungskräfte und Beschäftigte im Zentrum, Zuwendungsgeber und Versorgungsbeteiligte in Bochum sowie externe Fachöffentlichkeit) einbezog [4]. Vom 24.04.2017 bis zum 15.02.2019 wurden 4789 Erhebungsbögen ausgegeben. 3518 Personen (73,5 %) willigten ein, an der Evaluation teilzunehmen, 41.041 Leistungen wurden erfasst. Vulnerable Gruppen sind unterrepräsentiert, da sie häufig Bedenken hatten teilzunehmen. Ziel der Evaluation war, nicht nur die medizinischen und psychosozialen Angebote und ihre Nutzung zu bewerten, sondern auch, wie sich diese und die Niederschwelligkeit des WIR auf Akzeptanz und Zufriedenheit der Nutzer*innen und Nutzung der Angebote auswirken. Und nicht zuletzt sollte evaluiert werden, wie das Zusammenspiel zwischen den Institutionen funktioniert und welche Vorteile für Patient*innen/Klient*innen darin liegen. Im Evaluationsbericht heißt es zu der Zusammenarbeit der unterschiedlichen Einrichtungen im WIR: „Die gezeigten Daten lassen erkennen, dass sich die Klientel der Einrichtungen mitunter stark unterscheidet.“ [4, S. 45]. Durch die Kooperation werden häufiger Frauen (27,7 %) und Heterosexuelle (56,4 %) als in der Ambulanz allein erreicht.

Zudem stammen die Klient*innen aus unterschiedlichen Regionen (Tab. 1; [4]).

**Table 1:** 

Merkmale	Erstkontakt über … (Angaben in %)			
	Ambulanz (*n* = 1739)	Aidshilfe (*n* = 247)	Gesundheitsamt (*n* = 1530)	Gesamt^a^ (*n* = 3518)
*Ø Alter (Jahre)*	41,5	37,2	30,3	36,3
*Geschlecht*				
Männlich	80,4	90,2	59,8	72,1
Weiblich	19,4	9,8	40,0	27,7
Divers	0,2	0,0	0,2	0,2
*Sexuelle Orientierung*				
Heterosexuell	46,9	19,0	73,3	56,4
MSM	40,7	68,4	15,4	31,6
Lesbisch	0,3	0,0	0,6	0,4
Bisexuell	8,2	9,7	8,8	8,6
*Personen mit max. 9 Jahren Schule*	10,2	6,6	2,2	6,5
*Personen mit eingeschränkten Verständigungsmöglichkeiten*	5,8	5,7	1,4	3,9
*Wohnort*				
Bochum	30,1	41,8	62,0	44,9
Sonstiges Ruhrgebiet	42,2	40,6	24,7	34,4
Sonstiges NRW	24,0	15,6	10,7	17,6
Anderes Bundesland	2,3	0,8	1,6	1,9
Anderes Land	1,3	1,2	1,0	1,2

*MSM* Männer, die Sex mit Männern haben, *NRW* Nordrhein-Westfalen

^a^ Die Klientel der Einrichtungen Madonna, pro familia sowie Rosa Strippe sind aufgrund geringer Fallzahlen nicht einzeln benannt, aber im Gesamt berücksichtigt

Die Klientel des GA im WIR ist jünger, häufiger weiblich und heterosexuell und hat eine höhere Schulbildung. Die AH erreicht erwartungsgemäß vorwiegend Männer, die Sex mit Männern haben (MSM). Die Ambulanz hat einen Anteil von 46,9 % Heterosexuellen, 80,4 % männlichen Besuchern. Deutliche Unterschiede gibt es beim Einzugsgebiet. Während sich im GA überwiegend Bochumer Bürger*innen vorstellten (62,0 %), umfasst das Einzugsgebiet der Ambulanz und der AH ganz NRW (Tab. 1). Gründe, das WIR aufzusuchen (Mehrfachnennungen möglich), waren: Information/Beratung 17,8 %; 29,1 % waren längerfristig im WIR in Behandlung; 67,3 % wollten sich auf eine oder mehrere STI testen lassen [4].

**Figure 2:**
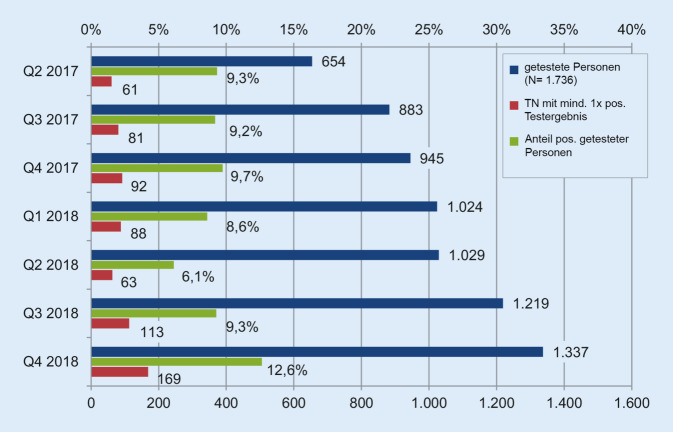

Mit steigender Zahl der TN-Testungen (645 auf 1337 von 2017 bis 2018) stieg die quantitative und auch die relative Rate der positiven Testergebnisse der TN mit einem oder mehreren positiven STI-Tests von 9,3 % auf 12,6 % (Abb. 2). Dies deutet auf einen Anstieg der STI-Prävalenz hin.

Bei 810 getesteten HIV-positiven Patient*innen lag die STI-Co-Infektionsrate insgesamt bei 25,3 %. Davon wurden im WIR über 96,5 % behandelt, bei 79,3 % Behandlungskontrollen gemacht, der Therapieerfolg lag im Durchschnitt bei 95,5 % (Tab. 2).

**Table 2:** 

Gesamt	Getestete Personen	Positiv getestet^a^	In Behandlung^a^	Behandlungskontrolle^a^	Erfolgreich abgeschlossen^a^
	810	205 (25,3 %)	198 (96,5 %)	157 (79,3 %)	150 (95,5 %)
Syphilis	809	68 (8,4 %)	66 (97,1 %)	56 (84,8 %)	49 (87,5 %)
Chlamydien	615	89 (14,5 %)	89 (100 %)	64 (71,9 %)	64 (100 %)
Gonorrhö	615	95 (15,4 %)	92 (96,8 %)	66 (71,7 %)	65 (98,5 %)
Mykoplasmen	614	69 (11,2 %)	66 (95,7 %)	52 (78,8 %)	47 (90,4 %)
Trichomonaden	614	6 (0,9 %)	6 (100 %)	1 (16,7 %)	1 (100 %)

^a^ Prozentangaben in Klammern beziehen sich auf die in der Spalte links daneben genannte Anzahl getesteter Personen

**Figure 3:**
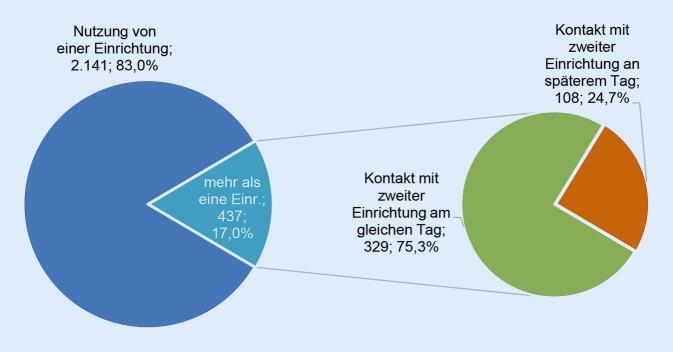

Wie sich die Grundwerte Wertschätzung, niedrigschwellig erreichbare Angebote, kombiniert mit medizinischer Expertise, auf die Klient*innen auswirken, zeigt deren Zufriedenheit, 90 % würden das WIR weiterempfehlen. Auszug aus dem Evaluationsbericht: „Tatsächlich waren 90 % der nachbefragten Klient*innen zufrieden oder sehr zufrieden mit dem WIR. Über 90 % der Befragten fühlten sich zudem respektiert, einige lobten die akzeptierende und wertschätzende Ansprache. Klient*innen berichteten, dass das Personal niemanden verurteilt, zuvorkommend ist und sich aktiv kümmert – auf fachlich hohem Niveau“ [4, S. 8]. Die 3 Hauptpartner, Ambulanz, AH, GA, haben eine unterschiedliche Gewichtung ihrer Zielgruppen, sodass Synergien aus der Ergänzung dieser 3 entstehen, die sich auch in der Überleitung von Klienten zwischen den Institutionen zeigt: 17 % der Besucher*innen nutzten mindestens 2 Einrichtungen des WIR, davon 75,3 % noch am selben Tag (Abb. 3). Dies ist bei positiven HIV/STI-Testergebnissen für eine sofortige medizinische Behandlung sowie psychotherapeutische Intervention wichtig. Letztere wird von mehr Männern als Frauen in Anspruch genommen, da Männer häufiger Infektionen mit HIV und anderen STI haben sowie Drogen oder Chemsex nutzen. Ebenfalls haben MSM meist einen komplexeren Beratungsbedarf [4].

### Studien und (Teil‑)Projekte

Aus der Arbeit im WIR ergeben sich Fragestellungen, die nach Möglichkeit Studien und (Teil‑)Projekte zur Folge haben. Diese sind aufgrund der Patient*innen‑/Klient*innenstruktur, der aufsuchenden Arbeit der HA und der unterschiedlichen Institutionen im WIR mit ihren unterschiedlichen Schwerpunkten sowie der externen Vernetzungen möglich. Die Ergebnisse fließen in die laufende Arbeit ein [16].

#### PreYoungGo-Studie.

Die Studie untersuchte die STI-Prävalenz, -Versorgung und das -Wissen bei jungen Erwachsenen. Bei 7,7 % der TN wurden *Chlamydia trachomatis* (CT) und bei 5,5 % *Neisseria gonorrhoeae* (NG) nachgewiesen (1,8 % Doppelinfektionen). Direkt am Tag der Diagnose wurden 70 % der TN behandelt. Erst auf Nachfrage gaben 13,2 % unspezifische Symptome vor Wochen an. Vor der Studienteilnahme hatten sich 59,9 % mindestens einmal auf HIV, 39,7 % auf CT, 20,6 % auf NG und 21,3 % auf Syphilis testen lassen. 47 % der Frauen hatten bereits Analverkehr [17].

#### PLUS-Initiative.

Das Projekt zur Versorgung von Drogennutzenden mit Fokus auf Hepatitis C (HCV; [18]) ermöglicht eine enge Vernetzung vor Ort (z. B. Krisenhilfe, WIR). Es konnte bisher (2020) bei allen 69 Behandelten eine HCV-Elimination erreicht werden (nicht publizierte Daten, interner WIR Jahresbericht).

#### HIV-PrEP-Versorgung in intersektoraler Zusammenarbeit.

Die Studie beschreibt die Nutzer der PrEP, die Veränderung des sexuellen Risikoverhaltens sowie Auswirkungen auf STI [19]. Das durchschnittliche Alter lag bei 38 Jahren, 98,6 % waren MSM mit hohem Bildungsstatus. Die durchschnittliche Partnerzahl in den letzten 6 Monaten stieg an, die Kondomnutzung sank signifikant von 26 % auf 7 %. Innerhalb der ersten 4 Monate nach PrEP-Beginn traten 44 STI, bei 34 Patienten aber keine HIV-Infektion auf. Über 370 PrEP-Nutzer – fast ausschließlich MSM – wurden bis April 2020 im WIR betreut.

#### GutVernetzt-Projekt.

Um die Versorgung im ländlichen Raum zu verbessern, hat das WIR gemeinsam mit dem Verband der AIDS-Koordinator*innen NRW e. V., den GA der Kreise Recklinghausen, Münster und Borken, der Fachstelle für Sexualität und Gesundheit – Aids-Hilfe Münster, der Evangelischen Frauenhilfe in Westfalen mit der Beratungsstelle Tamar-Münsterland und dem Streetwork-Projekt „Marischa“, GA Münster ein Vernetzungsprojekt für ländliche Standorte gestartet, unterstützt durch das Ministerium für Arbeit, Gesundheit und Soziales (MAGS) NRW [20].

## Diskussion

Das WIR – Walk In Ruhr, Zentrum für sexuelle Gesundheit und Medizin, war das erste integrierte Versorgungsangebot in Deutschland, das Prävention, Beratung, breite medizinische Behandlung, den Öffentlichen Gesundheitsdienst (ÖGD) und Selbsthilfe unter einem Dach vereinte [4]. Kontinuierlich werden die psychosozialen und -therapeutischen Angebote im WIR weiter ausgebaut. Die räumliche Nähe der beteiligten Institutionen im Zentrum „unter einem Dach“ ermöglicht und fördert fallbezogene Zusammenarbeit und aufeinander abgestimmte, sich ergänzende Präventions‑, Test- und Versorgungswege. Zeitnahe Überleitungen zu medizinischer, psychosozialer oder -therapeutischer Betreuung sind dadurch möglich. Infektionen oder Co-Infektionen werden schneller einer Behandlung zugeführt sowie psychosozialer Interventionsbedarf schneller erkannt und angeboten. Die Klientel des WIR ist heterogen, durch die Angebote aufsuchender, settingbezogener Arbeit werden auch schwer erreichbare, meist unterversorgte Menschen gezielt angesprochen [18, 21].

Das Konstrukt des Bochumer Zentrums aus freien Trägern, Krankenhausträgern und ÖGD gewährleistet ein breites Angebot, das sich an die Gesamtbevölkerung richtet, direkte Behandlung von HIV, Hepatitis und anderen STI bietet und notwendige stationäre Versorgung durch die unmittelbare Anbindung an das Krankenhaus ermöglicht. Dass die Teamarbeit der Akteure bei Test, Beratung, Behandlung unter einem Dach fruchtet, beweist die Tatsache, dass immerhin ein Sechstel der Klient*innen mehr als eine Institution am selben Tag nutzen, häufig aufgrund komplexer Probleme [4]. Um diese Entwicklung zu ermöglichen, war zum einen die räumliche Nähe notwendig und zum anderen wichtig, dass die beteiligten Partnereinrichtungen voneinander lernen, eine gemeinsame Sprache finden und zusammenwachsen – ein Prozess, der in wöchentlichen Teambesprechungen gefördert wird. Dies gelingt innerhalb der im Zentrum ansässigen Partner sehr gut, ist aber mit den nur stundenweise im Zentrum arbeitenden Partnern schwieriger.

Die Zahl der Nutzer*innen des WIR hat sich in 4 Jahren vervierfacht, von anfänglich 400 auf rund 1600 monatlich im Jahr 2020, dies trotz der Coronapandemie. Es ist sukzessive gelungen, verstärkt jüngere Klient*innen, mehr Frauen und auch mehr Heterosexuelle anzusprechen [4, 17]. Nur in Teilen ist dies bisher bei bildungsfernen Menschen, Flüchtlingen oder Menschen mit Behinderungen gelungen. Um hier mehr Dynamik zu erreichen, ist ein umfangreiches Projekt zu sexueller Gesundheit, ausschließlich in vulnerablen Gruppen, geplant.

Die Zahl der HA im WIR hat sich von anfänglich 2 auf zurzeit 4 erhöht, für das Jahr 2021 ist ein Ausbau auf 6 geplant. Grund ist, dass HA an immer mehr Schnittstellen arbeiten, die die besondere Nähe zu Klient*innen voraussetzen. Hinzu kommt die aufsuchende präventive Arbeit, mit dem Ziel, den Übergang von Prävention zu Testung und ggf. medizinischer und psychosozialer Versorgung zu bahnen. Zitat: „Die Evaluationsergebnisse belegen, dass durch die räumliche Zusammenfassung der Leistungserbringenden und vor allem durch die fallbezogene Zusammenarbeit, aufeinander abgestimmte Präventions-, Test- und Versorgungsangebote gefördert und Übertragungen und Ko-Infektionen frühzeitig erkannt und behandelt werden konnten. Das Ziel einer sektorübergreifenden Ausrichtung wird im WIR erreicht und dabei eine heterogene Klientel erreicht. Der Versorgungsbeitrag des Zentrums ist groß und hat sich im Untersuchungszeitraum weiter gesteigert. Dazu trugen maßgeblich die o. g. Präventionsangebote, bspw. in Schulen, Schwulenszene oder Swingerclubs, bei“ [4, S. 180]. Die im Zentrum eigens entwickelte curriculare Ausbildung für HA ist zwar gewährleistet, jedoch mangelt es an Zeit zur Reflexion und Weiterentwicklung der Angebote.

Die Rückläufe und Nutzerdaten zu den im WIR entwickelten Angeboten Online-HIV/STI-Risikotest [14], Partner*innenbenachrichtigung [15] und Probenselbstentnahmekit sind unterschiedlich. Während der Onlinetest auf enormen Zuspruch (rund 22.000 Nutzer*innen) stößt, wird die anonyme Partner*innenbenachrichtigung weniger genutzt. Es müssen Strategien entwickelt werden, um die Nutzung zu steigern, da sie ein wichtiges Instrument zur Reduktion von Infektionsketten ist. Im Zuge der Coronaschnelltestpublizität ist auch zu überlegen, ob und wie eine größere Zahl von Schnelltesten – gerade bei symptomlosen STI – die Behandlungszahlen erhöhen und die Neuinfektionen verringern könnte. Die Bemühungen, dass mehr Klient*innen im WIR Impfungen wahrnehmen – eine wichtige Stellschraube, um STI und Folgekomplikationen zu verhindern und die Kosten für das Gesundheitssystem zu reduzieren –, fruchten zwar, jedoch ist das Ziel, die Impfquote mit Kooperationspartnern aus unterschiedlichen Bereichen zu steigern.

Prävention durch Therapie gelingt bzgl. HIV zu 98 %. Sehr breit und effektiv sind die Aufklärung, Verschreibung und Therapiekontrolle zur PrEP angenommen worden [19]. Durch risikoadaptierte Testungen können nicht nur STI-Neuinfektionen, sondern auch HIV-Co-Infektionen früher behandelt werden [4, 16]. Zitat Evaluationsbericht S. 4: „Die Analyse der Laborergebnisse zeigt über die Zeit, dass trotz der Testung von immer mehr Personen die Positivrate nicht abfiel, sondern im Gegenteil leicht stieg. Die höhere Quote positiver STI-Testergebnisse kann als Indiz für die Relevanz und Treffsicherheit einer risikoadaptierten Testung betrachtet werden.“

Das WIR hat, naturgemäß in seinem ganzheitlichen Ansatz von Versorgung zu sexueller Gesundheit begründet, ein großes Angebot an psychosozialen und -therapeutischen Leistungen. Ziele sind die Stärkung des Bewusstseins für die Eigenverantwortung und die Chance zur eigenen sexuellen Gesundheit, des Selbstwerts und der Selbstachtung (Empowerment) sowie die Förderung von risikoadaptiertem Verhalten und Therapietreue.

Es ist bisher nicht gelungen, Leistungen der „sprechenden Medizin“ in die Regelversorgung einzubinden. Folglich muss viel Zeit für das Einwerben von Drittmitteln, Projekten und Studien sowie Spenden aufgebracht werden. Der erste Mut machende Erfolg ist ein Selektivvertrag, der zuerst mit der Knappschaft-Bahn-See und dann mit der Viactiv Krankenversicherung abgeschlossen werden konnte. Er hat eine „Qualitätspauschale“ zum Inhalt, die umfassende Beratung und Behandlung zu sexueller Gesundheit sichert. Ähnliche Verträge mit möglichst vielen Versicherern werden angestrebt. Ein weiterer Teilerfolg ist, dass das Spektrum der abrechenbaren Leistungen seit Herbst 2020 von der Kassenärztlichen Vereinigung auf psychotherapeutische Leistungen und Tumorvorsorgeuntersuchungen erweitert werden konnte.

## Fazit

Bedenkt man die Ergebnisse von 4 Jahren Erfahrung im WIR, so gilt es festzuhalten, dass ein Konzept integrierter Prävention, Beratung, Testung und Behandlung gelingt, wenn medizinische und beratende Kompetenzen miteinander einhergehen, wenn Handlungen sich an Lebenswelten orientieren und eine wertschätzende Haltung allen Individuen entgegengebracht wird. Insofern wäre die Ausweitung ähnlicher Angebote, z. B. in Kooperationen, nicht nur für die sexuelle Gesundheit von Menschen, sondern auch aus gesundheitsökonomischen Gründen anzustreben.

## Footnotes

1 (https://www.unaids.org).
