# Erratum zu: Sexuelle Gesundheit und Medizin im WIR – Walk In Ruhr: Vorstellung des Zentrums und Ergebnisse der Evaluation

**DOI:** 10.1007/s00103-022-03522-1

**Published:** 2022-03-29

**Authors:** Anja Potthoff, Adriane Skaletz-Rorowski, Sandeep Nambiar, Wiltrud Knebel-Brockmeyer, Andre Kasper, Janet Wach, Arne Kayser, Britta Köhler, Norbert H. Brockmeyer

**Affiliations:** 1 Zentrum für Sexuelle Gesundheit und Medizin, Walk In Ruhr (WIR), Große Beckstraße 12, 44787 Bochum, Deutschland; 2 Interdisziplinäre Immunologische Ambulanz, Zentrum für Sexuelle Gesundheit und Medizin, Klinik für Dermatologie, Venerologie und Allergologie, Ruhr Universität Bochum, Bochum, Deutschland; 3 Gesundheitsamt Bochum, Bochum, Deutschland; 4 Aidshilfe Bochum e. V., Bochum, Deutschland

**Erratum zu:**

**Bundesgesundheitsbl 2021**

10.1007/s00103-021-03382-1

Die ursprüngliche Originalfassung wurde angepasst, um die Prozesse der externen Projektevaluation angemessener und genauer zu beschreiben.

Dabei wurde in der Zusammenfassung verdeutlicht, welche Zahlen dem nicht veröffentlichten internen Walk In Ruhr(WIR)-Jahresbericht entnommen wurden.

Das Ziel und die Methode der Arbeit wurden präzisiert. Die Autoren haben die Schlussfolgerung geprüft und durch entsprechende Ergänzungen und Literatur im Haupttext untermauert.

Die Ergebnisse des Evaluationsberichtes wurden dabei deutlich von den anderen Daten des WIR getrennt dargestellt. Die entsprechenden Änderungen finden Sie im Update.

Der Originalbeitrag wurde korrigiert.

